# (*E*)-*N*′-(2,3-Dihy­droxy­benzyl­idene)-4-meth­oxy­benzohydrazide

**DOI:** 10.1107/S1600536811045740

**Published:** 2011-11-12

**Authors:** Premrudee Promdet, Jirapa Horkaew, Suchada Chantrapromma, Hoong-Kun Fun

**Affiliations:** a Crystal Materials Research Unit, Department of Chemistry, Faculty of Science, Prince of Songkla University, Hat-Yai, Songkhla 90112, Thailand; b X-ray Crystallography Unit, School of Physics, Universiti Sains Malaysia, 11800 USM, Penang, Malaysia

## Abstract

The mol­ecule of the title benzohydrazide derivative, C_15_H_14_N_2_O_4_, is twisted and exists in a *trans* conformation with respect to the C=N double bond. The dihedral angle between the benzene rings is 56.86 (9)° and the C atom of the meth­oxy group deviates slightly [C—O—C—C = −10.4 (3)°] from its attached benzene ring. An intra­molecular O—H⋯N hydrogen bond generates an *S*(6) ring. In the crystal, mol­ecules are linked by N—H⋯O and bifurcated N—H⋯(O,O) hydrogen bonds, as well as weak C—H⋯O inter­actions, into two-dimensional networks lying parallel to the *bc* plane. A weak C—H⋯π inter­action also occurs.

## Related literature

For background to benzohydrides and related structures, see: Fun *et al.* (2011 ▶); Horkaew *et al.* (2011 ▶). For related structures, see: Han & Zhao (2010 ▶); Li & Ban (2009 ▶). For reference bond-length data, see: Allen *et al.* (1987 ▶). For graph-set theory, see: Bernstein *et al.* (1995 ▶).
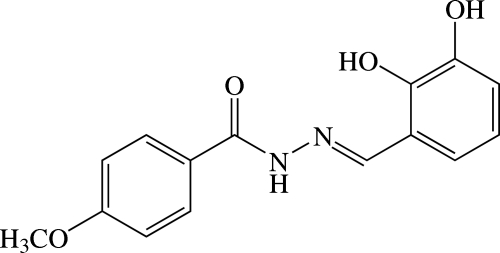


## Experimental

### 

#### Crystal data

C_15_H_14_N_2_O_4_
*M* *_r_* = 286.28
Monoclinic, 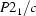

*a* = 11.6242 (15) Å
*b* = 9.7516 (13) Å
*c* = 12.6465 (16) Å
β = 96.409 (2)°
*V* = 1424.6 (3) Å^3^
*Z* = 4
Mo *K*α radiation
μ = 0.10 mm^−1^
*T* = 297 K
0.34 × 0.22 × 0.08 mm

#### Data collection

Bruker SMART APEXII CCD diffractometer
Absorption correction: multi-scan (*SADABS*; Bruker, 2009 ▶) *T*_min_ = 0.967, *T*_max_ = 0.992
13930 measured reflections
3768 independent reflections
2203 reflections with *I* > 2σ(*I*)
*R*_int_ = 0.037

#### Refinement

*R*[*F* ^2^ > 2σ(*F* ^2^)] = 0.046
*wR*(*F* ^2^) = 0.136
*S* = 1.02
3768 reflections
203 parameters
H atoms treated by a mixture of independent and constrained refinement
Δρ_max_ = 0.20 e Å^−3^
Δρ_min_ = −0.18 e Å^−3^

### 

Data collection: *APEX2* (Bruker, 2009 ▶); cell refinement: *SAINT* (Bruker, 2009 ▶); data reduction: *SAINT*; program(s) used to solve structure: *SHELXTL* (Sheldrick, 2008 ▶); program(s) used to refine structure: *SHELXTL*; molecular graphics: *SHELXTL*; software used to prepare material for publication: *SHELXTL* and *PLATON* (Spek, 2009 ▶).

## Supplementary Material

Crystal structure: contains datablock(s) global, I. DOI: 10.1107/S1600536811045740/hb6485sup1.cif

Structure factors: contains datablock(s) I. DOI: 10.1107/S1600536811045740/hb6485Isup2.hkl

Supplementary material file. DOI: 10.1107/S1600536811045740/hb6485Isup3.cml

Additional supplementary materials:  crystallographic information; 3D view; checkCIF report

## Figures and Tables

**Table 1:** Hydrogen-bond geometry (Å, °) *Cg*1 is the centroid of the C9–C14 ring.

*D*—H⋯*A*	*D*—H	H⋯*A*	*D*⋯*A*	*D*—H⋯*A*
O3—H1*O*3⋯N2	0.88 (3)	1.86 (3)	2.6279 (18)	146 (2)
O4—H1*O*4⋯O1^i^	0.92 (2)	1.75 (2)	2.6577 (19)	170 (2)
N1—H1*N*1⋯O3^ii^	0.864 (19)	2.221 (19)	3.083 (2)	175.8 (17)
N1—H1*N*1⋯O4^ii^	0.864 (19)	2.481 (19)	2.961 (2)	115.8 (15)
C5—H5*A*⋯O2^iii^	0.93	2.50	3.413 (2)	166
C2—H2*A*⋯*Cg*1^iv^	0.93	3.00	3.539 (2)	119

Symmetry codes: (i) 
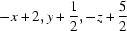
; (ii) 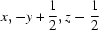; (iii) 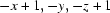; (iv) 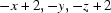.

**Fig. 1.**
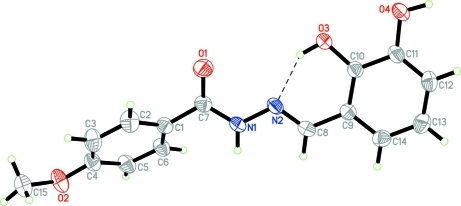
The molecular structure of the title compound, showing 40% probability displacement ellipsoids. Hydrogen bond is drawn as a dashed line.

**Fig. 2.**
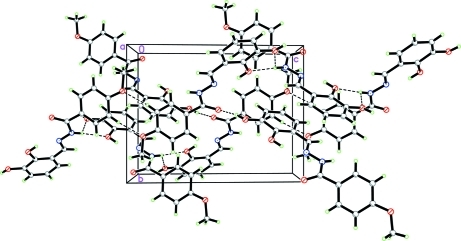
The crystal packing of the title compound viewed along the a-axis. Hydrogen bonds were drawn as dashed lines.

## supplementary crystallographic information

### Comment

Our on-going research on the biological activities of benzohydrazides containing the -CO-NH-N=CH- grouping has led us to synthesize the title compound (I) in order to compare its activity with other related compounds (Fun *et al.*, 2011; Horkaew *et al.*, 2011). Our results found that (I) exhibits interesting antibacterial and antioxidant activities which will be reported elsewhere with other related benzohydrazide derivatives. Herein the crystal structure of (I) is reported.

The molecule of the title benzohydrazide derivative (Fig. 1), C_15_H_14_N_2_O_4_, is twisted and exists in a *trans*-configuration with respect to the C8═N2 bond [1.280 (2) Å] and the torsion angle N1–N2–C8–C9 = 178.17 (15)°. The dihedral angle between the two benzene rings is 56.86 (9)°. The middle fragment is slightly twisted as indicated by the torsion angles O1–C7–N1–N2 = -0.8 (3)° and C7–N1–N2–C8 = 169.90 (16)°. The mean plane through this middle bridge (O1/C7/N1/N2/C8) makes the dihedral angles of 41.08 (11) and 16.45 (10)° with the planes of 4-methoxyphenyl and 2,3-dihydroxyphenyl rings, respectively. The two hydroxy groups of the 2,3-dihydroxyphenyl are co-planar with their attached benzene ring with the *r.m.s.* = 0.0214 (2) Å for the eight non H atoms. The methoxy group is slightly twisted from its attached benzene ring with the torsion angle C15–O2–C4–C3 = -10.4 (3)°. Bond distances of (I) are in normal range (Allen *et al.*, 1987) and are comparable with the related structures (Fun *et al.*, 2011; Han & Zhao, 2010; Li & Ban, 2009).

In the crystal packing (Fig. 2), the molecules are linked by N—H···O, and O—H···O hydrogen bonds, as well as with weak C—H···O interactions (Table 1), into two dimensional networks parallel to the *bc* plane. A C—H···π interaction was also presented (Table 1).

### Experimental

4-Methoxybenzohydrazide (2 mmol, 0.33 g) was dissolved in ethanol (10 ml) and a solution of 2,3-dihydroxybenzaldehyde (2 mmol, 0.28 g) in ethanol (10 ml) was then slowly added to it. The mixture was refluxed for around 5 hr. The solution was then cooled to room temperature and left to evaporate in air. The yellow solid product that appeared was collected by filtration and washed with ethanol and dried in air. Yellow blocks of the title compound were obtained after recrystalization from methanol by the slow evaporation of the solvent at room temperature after several days, Mp. 502-503 K.

### Refinement

Amide and hydroxy H atoms were located from the difference maps and refined isotropically. The remaining H atoms were positioned geometrically and allowed to ride on their parent atoms, with d(C-H) = 0.93 Å for aromatic and CH and 0.96 Å for CH_3_ atoms. The *U*_iso_ values were constrained to be 1.5*U*_eq_ of the carrier atom for methyl H atoms and 1.2*U*_eq_ for the remaining H atoms. A rotating group model was used for the methyl groups. The highest residual electron density peak is located at 0.72 Å from C9 and the deepest hole is located at 1.02 Å from C3.

### Crystal data

**Table:**

C_15_H_14_N_2_O_4_	*F*(000) = 600
*M**_r_* = 286.28	*D*_x_ = 1.335 Mg m^−^^3^
Monoclinic, *P*2_1_/*c*	Melting point = 502–503 K
Hall symbol: -P 2ybc	Mo *K*α radiation, λ = 0.71073 Å
*a* = 11.6242 (15) Å	Cell parameters from 3768 reflections
*b* = 9.7516 (13) Å	θ = 2.6–29.0°
*c* = 12.6465 (16) Å	µ = 0.10 mm^−^^1^
β = 96.409 (2)°	*T* = 297 K
*V* = 1424.6 (3) Å^3^	Block, yellow
*Z* = 4	0.34 × 0.22 × 0.08 mm

### Data collection

**Table:**

Bruker SMART APEXII CCD diffractometer	3768 independent reflections
Radiation source: fine-focus sealed tube	2203 reflections with *I* > 2σ(*I*)
graphite	*R*_int_ = 0.037
Detector resolution: 8.33 pixels mm^-1^	θ_max_ = 29.0°, θ_min_ = 2.6°
ω scans	*h* = −14→15
Absorption correction: multi-scan (*SADABS*; Bruker, 2009)	*k* = −13→12
*T*_min_ = 0.967, *T*_max_ = 0.992	*l* = −17→17
13930 measured reflections	

### Refinement

**Table:**

Refinement on *F*^2^	Primary atom site location: structure-invariant direct methods
Least-squares matrix: full	Secondary atom site location: difference Fourier map
*R*[*F*^2^ > 2σ(*F*^2^)] = 0.046	Hydrogen site location: inferred from neighbouring sites
*wR*(*F*^2^) = 0.136	H atoms treated by a mixture of independent and constrained refinement
*S* = 1.02	*w* = 1/[σ^2^(*F*_o_^2^) + (0.0547*P*)^2^ + 0.2931*P*] where *P* = (*F*_o_^2^ + 2*F*_c_^2^)/3
3768 reflections	(Δ/σ)_max_ = 0.001
203 parameters	Δρ_max_ = 0.20 e Å^−^^3^
0 restraints	Δρ_min_ = −0.18 e Å^−^^3^

### Special details

**Table:**

Geometry. All esds (except the esd in the dihedral angle between two l.s. planes) are estimated using the full covariance matrix. The cell esds are taken into account individually in the estimation of esds in distances, angles and torsion angles; correlations between esds in cell parameters are only used when they are defined by crystal symmetry. An approximate (isotropic) treatment of cell esds is used for estimating esds involving l.s. planes.
Refinement. Refinement of F^2^ against ALL reflections. The weighted R-factor wR and goodness of fit S are based on F^2^, conventional R-factors R are based on F, with F set to zero for negative F^2^. The threshold expression of F^2^ > 2sigma(F^2^) is used only for calculating R-factors(gt) etc. and is not relevant to the choice of reflections for refinement. R-factors based on F^2^ are statistically about twice as large as those based on F, and R- factors based on ALL data will be even larger.

### Fractional atomic coordinates and isotropic or equivalent isotropic displacement parameters (Å^2^)

**Table:**

	*x*	*y*	*z*	*U*_iso_*/*U*_eq_	
O1	0.76198 (12)	0.03025 (16)	1.01358 (9)	0.0644 (4)	
O2	0.52798 (13)	−0.17500 (15)	0.56417 (11)	0.0716 (4)	
O3	0.95302 (12)	0.31125 (15)	1.18591 (10)	0.0573 (4)	
H1O3	0.919 (2)	0.266 (3)	1.131 (2)	0.105 (9)*	
O4	1.07704 (14)	0.45742 (15)	1.33066 (10)	0.0655 (4)	
H1O4	1.134 (2)	0.491 (2)	1.380 (2)	0.092 (8)*	
N1	0.86300 (14)	0.15397 (15)	0.90461 (11)	0.0472 (4)	
H1N1	0.8895 (16)	0.1593 (18)	0.8436 (15)	0.050 (5)*	
N2	0.92453 (13)	0.21946 (15)	0.98936 (10)	0.0457 (4)	
C1	0.71751 (15)	−0.00089 (18)	0.82727 (13)	0.0432 (4)	
C2	0.68810 (17)	−0.13814 (19)	0.82957 (15)	0.0574 (5)	
H2A	0.7107	−0.1896	0.8903	0.069*	
C3	0.62568 (18)	−0.2001 (2)	0.74317 (17)	0.0621 (5)	
H3A	0.6080	−0.2930	0.7453	0.075*	
C4	0.58959 (16)	−0.12397 (19)	0.65375 (14)	0.0516 (5)	
C5	0.61520 (18)	0.0146 (2)	0.65158 (14)	0.0548 (5)	
H5A	0.5883	0.0672	0.5926	0.066*	
C6	0.68036 (17)	0.07452 (19)	0.73656 (13)	0.0514 (5)	
H6A	0.6999	0.1668	0.7334	0.062*	
C7	0.78258 (15)	0.06096 (18)	0.92317 (12)	0.0445 (4)	
C8	1.01140 (16)	0.29144 (17)	0.96886 (13)	0.0451 (4)	
H8A	1.0311	0.2950	0.8996	0.054*	
C9	1.07959 (15)	0.36759 (16)	1.05231 (12)	0.0417 (4)	
C10	1.04835 (15)	0.37551 (17)	1.15543 (13)	0.0426 (4)	
C11	1.11564 (16)	0.45196 (17)	1.23312 (13)	0.0464 (4)	
C12	1.21434 (17)	0.51668 (18)	1.20748 (15)	0.0525 (5)	
H12A	1.2599	0.5662	1.2593	0.063*	
C13	1.24602 (18)	0.50867 (19)	1.10575 (16)	0.0569 (5)	
H13A	1.3128	0.5527	1.0894	0.068*	
C14	1.17932 (17)	0.43587 (18)	1.02824 (14)	0.0512 (5)	
H14A	1.2006	0.4321	0.9596	0.061*	
C15	0.5152 (2)	−0.3199 (2)	0.5548 (2)	0.0828 (7)	
H15A	0.4773	−0.3422	0.4856	0.124*	
H15B	0.4697	−0.3528	0.6083	0.124*	
H15C	0.5902	−0.3624	0.5642	0.124*	

### Atomic displacement parameters (Å^2^)

**Table:**

	*U*^11^	*U*^22^	*U*^33^	*U*^12^	*U*^13^	*U*^23^
O1	0.0620 (9)	0.0923 (11)	0.0369 (6)	−0.0148 (8)	−0.0038 (6)	0.0134 (6)
O2	0.0770 (11)	0.0673 (9)	0.0646 (9)	−0.0017 (8)	−0.0194 (7)	−0.0185 (7)
O3	0.0611 (9)	0.0729 (9)	0.0388 (6)	−0.0241 (7)	0.0097 (6)	−0.0122 (6)
O4	0.0805 (11)	0.0773 (10)	0.0381 (7)	−0.0266 (8)	0.0033 (7)	−0.0101 (6)
N1	0.0545 (10)	0.0569 (9)	0.0299 (7)	−0.0064 (7)	0.0029 (6)	−0.0053 (6)
N2	0.0505 (9)	0.0523 (8)	0.0330 (7)	−0.0019 (7)	−0.0011 (6)	−0.0064 (6)
C1	0.0434 (10)	0.0478 (9)	0.0371 (8)	0.0021 (8)	−0.0017 (7)	0.0014 (7)
C2	0.0612 (13)	0.0513 (11)	0.0557 (11)	0.0040 (9)	−0.0112 (9)	0.0120 (8)
C3	0.0671 (14)	0.0444 (10)	0.0707 (12)	−0.0024 (9)	−0.0107 (10)	0.0010 (9)
C4	0.0473 (11)	0.0560 (11)	0.0494 (10)	0.0029 (9)	−0.0036 (8)	−0.0099 (8)
C5	0.0683 (13)	0.0549 (11)	0.0381 (9)	0.0039 (9)	−0.0073 (8)	0.0027 (8)
C6	0.0685 (13)	0.0455 (10)	0.0381 (8)	−0.0016 (9)	−0.0036 (8)	0.0023 (7)
C7	0.0441 (10)	0.0522 (10)	0.0356 (8)	0.0049 (8)	−0.0025 (7)	0.0041 (7)
C8	0.0537 (11)	0.0469 (9)	0.0348 (8)	0.0037 (8)	0.0054 (7)	−0.0022 (7)
C9	0.0448 (10)	0.0406 (9)	0.0391 (8)	0.0025 (7)	0.0021 (7)	0.0012 (7)
C10	0.0448 (10)	0.0431 (9)	0.0392 (8)	−0.0026 (7)	0.0013 (7)	0.0008 (7)
C11	0.0555 (12)	0.0435 (9)	0.0383 (8)	−0.0038 (8)	−0.0029 (8)	0.0006 (7)
C12	0.0561 (12)	0.0438 (10)	0.0545 (10)	−0.0082 (9)	−0.0080 (9)	0.0023 (8)
C13	0.0530 (12)	0.0523 (11)	0.0654 (12)	−0.0090 (9)	0.0066 (9)	0.0067 (9)
C14	0.0554 (12)	0.0507 (10)	0.0487 (10)	−0.0017 (9)	0.0110 (9)	0.0045 (8)
C15	0.0923 (19)	0.0771 (16)	0.0788 (15)	−0.0244 (14)	0.0085 (13)	−0.0273 (13)

### Geometric parameters (Å, °)

**Table:**

O1—C7	1.231 (2)	C4—C5	1.385 (3)
O2—C4	1.365 (2)	C5—C6	1.374 (2)
O2—C15	1.424 (3)	C5—H5A	0.9300
O3—C10	1.365 (2)	C6—H6A	0.9300
O3—H1O3	0.88 (3)	C8—C9	1.451 (2)
O4—C11	1.360 (2)	C8—H8A	0.9300
O4—H1O4	0.91 (3)	C9—C10	1.394 (2)
N1—C7	1.342 (2)	C9—C14	1.400 (2)
N1—N2	1.3773 (18)	C10—C11	1.401 (2)
N1—H1N1	0.863 (19)	C11—C12	1.379 (3)
N2—C8	1.280 (2)	C12—C13	1.379 (3)
C1—C2	1.382 (3)	C12—H12A	0.9300
C1—C6	1.390 (2)	C13—C14	1.377 (3)
C1—C7	1.484 (2)	C13—H13A	0.9300
C2—C3	1.382 (3)	C14—H14A	0.9300
C2—H2A	0.9300	C15—H15A	0.9600
C3—C4	1.379 (3)	C15—H15B	0.9600
C3—H3A	0.9300	C15—H15C	0.9600
			
C4—O2—C15	118.09 (17)	N2—C8—C9	120.83 (15)
C10—O3—H1O3	108.7 (17)	N2—C8—H8A	119.6
C11—O4—H1O4	110.3 (16)	C9—C8—H8A	119.6
C7—N1—N2	119.26 (14)	C10—C9—C14	119.05 (16)
C7—N1—H1N1	121.5 (12)	C10—C9—C8	122.03 (16)
N2—N1—H1N1	117.5 (12)	C14—C9—C8	118.92 (15)
C8—N2—N1	116.72 (14)	O3—C10—C9	122.88 (15)
C2—C1—C6	118.34 (16)	O3—C10—C11	116.98 (15)
C2—C1—C7	118.75 (15)	C9—C10—C11	120.15 (16)
C6—C1—C7	122.85 (16)	O4—C11—C12	124.23 (16)
C3—C2—C1	121.08 (17)	O4—C11—C10	116.24 (16)
C3—C2—H2A	119.5	C12—C11—C10	119.52 (16)
C1—C2—H2A	119.5	C11—C12—C13	120.59 (17)
C4—C3—C2	119.86 (18)	C11—C12—H12A	119.7
C4—C3—H3A	120.1	C13—C12—H12A	119.7
C2—C3—H3A	120.1	C14—C13—C12	120.36 (18)
O2—C4—C3	124.61 (18)	C14—C13—H13A	119.8
O2—C4—C5	115.69 (17)	C12—C13—H13A	119.8
C3—C4—C5	119.71 (17)	C13—C14—C9	120.31 (17)
C6—C5—C4	120.02 (17)	C13—C14—H14A	119.8
C6—C5—H5A	120.0	C9—C14—H14A	119.8
C4—C5—H5A	120.0	O2—C15—H15A	109.5
C5—C6—C1	120.93 (17)	O2—C15—H15B	109.5
C5—C6—H6A	119.5	H15A—C15—H15B	109.5
C1—C6—H6A	119.5	O2—C15—H15C	109.5
O1—C7—N1	122.57 (16)	H15A—C15—H15C	109.5
O1—C7—C1	121.74 (17)	H15B—C15—H15C	109.5
N1—C7—C1	115.67 (14)		
			
C7—N1—N2—C8	169.90 (16)	C6—C1—C7—N1	−40.2 (2)
C6—C1—C2—C3	1.5 (3)	N1—N2—C8—C9	178.17 (15)
C7—C1—C2—C3	178.77 (19)	N2—C8—C9—C10	−5.6 (3)
C1—C2—C3—C4	−1.4 (3)	N2—C8—C9—C14	174.85 (16)
C15—O2—C4—C3	−10.4 (3)	C14—C9—C10—O3	−179.49 (16)
C15—O2—C4—C5	170.0 (2)	C8—C9—C10—O3	1.0 (3)
C2—C3—C4—O2	179.78 (19)	C14—C9—C10—C11	0.6 (3)
C2—C3—C4—C5	−0.6 (3)	C8—C9—C10—C11	−178.97 (16)
O2—C4—C5—C6	−177.74 (19)	O3—C10—C11—O4	−1.8 (2)
C3—C4—C5—C6	2.6 (3)	C9—C10—C11—O4	178.15 (16)
C4—C5—C6—C1	−2.6 (3)	O3—C10—C11—C12	178.61 (16)
C2—C1—C6—C5	0.5 (3)	C9—C10—C11—C12	−1.5 (3)
C7—C1—C6—C5	−176.62 (18)	O4—C11—C12—C13	−178.43 (18)
N2—N1—C7—O1	−0.8 (3)	C10—C11—C12—C13	1.1 (3)
N2—N1—C7—C1	177.88 (14)	C11—C12—C13—C14	0.0 (3)
C2—C1—C7—O1	−38.7 (3)	C12—C13—C14—C9	−0.9 (3)
C6—C1—C7—O1	138.5 (2)	C10—C9—C14—C13	0.6 (3)
C2—C1—C7—N1	142.68 (18)	C8—C9—C14—C13	−179.82 (17)

### Hydrogen-bond geometry (Å, °)

**Table:**

*Cg*1 is the centroid of the C9–C14 ring.

**Table:**

*D*—H···*A*	*D*—H	H···*A*	*D*···*A*	*D*—H···*A*
O3—H1O3···N2	0.88 (3)	1.86 (3)	2.6279 (18)	146 (2)
O4—H1O4···O1^i^	0.92 (2)	1.75 (2)	2.6577 (19)	170 (2)
N1—H1N1···O3^ii^	0.864 (19)	2.221 (19)	3.083 (2)	175.8 (17)
N1—H1N1···O4^ii^	0.864 (19)	2.481 (19)	2.961 (2)	115.8 (15)
C5—H5A···O2^iii^	0.93	2.50	3.413 (2)	166
C2—H2A···Cg1^iv^	0.93	3.00	3.539 (2)	119

Symmetry codes: (i) −*x*+2, *y*+1/2, −*z*+5/2; (ii) *x*, −*y*+1/2, *z*−1/2; (iii) −*x*+1, −*y*, −*z*+1; (iv) −*x*+2, −*y*, −*z*+2.
